# The Emergency Medicine Educational Community and the Supply Side of the Workforce

**DOI:** 10.5811/westjem.2022.11.58880

**Published:** 2022-12-28

**Authors:** David J. Carlberg

**Affiliations:** Georgetown University School of Medicine, Department of Emergency Medicine, Washington DC

## INTRODUCTION

Emergency medicine (EM) is at an inflection point: The specialty’s response to the projected workforce surplus will shape the specialty for decades. Emergency physician (EP) educators, especially residency and clerkship leadership, have a duty to address the pipeline into EM, specifically the marked growth of EM residency positions via program expansion and creation. The pipeline problem is complex: How does the specialty slow or reverse the growth of EM residency positions while so many emergency departments (ED) and EPs could provide exceptional education if given the opportunity?

The answers will be just as complex. They will require open, honest, and respectful discussion; and they will require nuance, introspection, and a focus on the greater good. Both the search for answers and the potential solutions will be uncomfortable:

- Established EM residency programs need to consider harms of expansion and potential benefits of contraction;
- Planned residencies need to weigh their impact on the greater EM community given the predicted oversaturated job market; and
- Professional organizations need to directly address the rapid expansion of EM residency positions.

If educators, clerkships, residency programs, and professional organizations take urgent and appropriately aggressive actions, potentially drastic workforce sequelae may be averted.

## DEFINING THE PROBLEM

### Residency Positions and the EM Workforce

The workforce numbers appear grim. Projections suggest an over-supply of 7,845 EPs by 2030.^1^ The marked increase in EM residency positions largely contributes to the over-supply. Even accounting for American Osteopathic Association EM residency programs transitioning to the Accreditation Council for Graduate Medical Education (ACGME) under the single accreditation system (SAS), EM postgraduate year-1 (PGY-1) positions increased from 2,056 to 2,840 (38%) between 2014–2021. Emergency medicine had the largest growth rate of PGY-1 positions among all medical specialties from 2014 to 2019, growing twice as fast as the overall number of residency positions across specialties.^2^ While many of these additional residency positions resulted from new programs opening, the expansion and contraction of established programs led to the creation of an estimated 129 additional EM PGY-1 positions from 2018 to 2022. Roughly 70 programs expanded while 13 contracted.^3^

The number of EM residency programs increased from 222 to 273 (23%) between 2014–2021 after accounting for the SAS.^2^ An average of nine new EM residency programs opened annually from 2016 to 2021, while an average of four programs opened annually between 1983–2015.^2^ Many of the new programs founded between 2013–2020 opened in states with a plethora of established programs. The number of programs in Florida nearly quadrupled (from 5 to 19), and the number of programs in Michigan and Ohio at least doubled (11 to 25 and 9 to 18, respectively).^4^

Of the 42 EM residency programs currently on initial ACGME accreditation, nearly a quarter (10) are accredited “with warning”; for comparison, only six of 236 programs on continued accreditation are accredited “with warning.”^5^

The proportion of EM education delivered at for-profit hospitals has also changed considerably. Before 2016, only 4% of new training sites were located at for-profit hospitals, but from 2016 to 2021, 37% of new sites were located at for-profit hospitals.^2^

### The 2022 Emergency Medicine Match

The data from the 2022 EM match was not much better. Of the 277 EM residency programs participating in the Match, 69 did not fill (25%), and of the 2,921 EM residency positions offered, 219 did not fill (7.5%). For comparison, there were 14 unfilled EM positions in the 2021 Match (0.5%).^6^

While EM programs that filled in 2021 ranked an average of 5.8 applicants per position, this year EM programs that filled ranked an average of 8.8 applicants per position. The only specialties that ranked more applicants per position in order to fill were internal medicine and pediatrics. This year only 1.1% of seniors graduating from allopathic medical schools applying solely to EM went unmatched, and the only specialties with lower unmatched rates were pediatrics and child neurology. Additionally, 25 EM residency programs did not fill more than half their quota of spots in 2022; three programs filled only one spot; and two programs did not fill any spots.^6^

Some EM educators believe that market forces will bring the supply side of the EM workforce back into balance, suggesting that the overall number of EM residency positions offered will decrease because of poor Match results.^7^ However, with 202 of the 217 available EM PGY-1 positions filling in the Supplemental Offer and Acceptance Program (SOAP), and with 57 of 67 EM residency programs participating in the SOAP completely filling,^6^ the power of market forces to reign in the expansion of EM residency positions is likely limited.

### Incentives to Create New Residency Positions

Hospitals and EDs may have financial incentives to develop new EM residency positions, especially when creating training programs at hospitals without established graduate medical education (GME) programs. In 2018, Medicare paid teaching hospitals an average of $171,000 per funded resident.^8^ While hospitals with GME programs in existence for more than five years are capped in their number of Medicare-funded trainees, hospitals with GME programs developed more recently do not yet have established trainee caps; so expanding the number of trainees increases Medicare funding.^8^ Additionally, if GME trainees are used to fulfill a service need, thereby reducing the demand for non-resident healthcare professionals, adding GME trainees is likely financially beneficial. A 2013 study estimated that adding an internal medicine resident to fulfill a service need could save a hospital $43,707 and that adding a cardiology fellow to fulfill a service need could save a hospital $151,694 before accounting for the additional Medicare and Medicaid funding those trainees could bring.^9^

## ADDRESSING THE PROBLEM

While identifying the multitude of factors underlying the rapid growth of EM residency positions is vitally important to developing and implementing solutions, attributing blame with a goal of punishment or retaliation prevents collaboration and removes the nuance necessary to find answers.

Potential solutions may not satisfy all stakeholders. Dr. Gillian Schmitz, president of the American College of Emergency Physicians (ACEP), said: “We need to start having some difficult conversations on how we control [residency] growth in a responsible manner and put the needs of the specialty ahead of any individual residency program’s best interests.”^10^ This message is important for all to hear: individual educators, residency program leadership, and national organizations. Individual programs may be disadvantaged by some solutions, not out of malicious intent, but for the betterment of the specialty. Conversely, if the community of EM educators demands solutions that make everyone happy, attempts to address the pipeline into EM will fail.

Finding and implementing solutions will take thoughtful and potentially aggressive action by EM educators, residency programs, clerkships, and national organizations.

### EM Educators

While EM faculty may feel sheltered from workforce issues because of a modicum of job security and some level of insulation provided by the academic orb, EM educators must still work to understand the problem, its scope, and potential solutions. Faculty should feel empowered to create the space and time necessary to respectfully discuss the problem and the uncertainty and angst surrounding it, both with colleagues and with learners.

Forums like the Council of Residency Directors in EM (CORD) list-serv or the Clerkship Directors in EM (CDEM) list-serv may be great venues for advancing the discussions, proposing potential solutions, and pushing one another to think outside the box. The power of the “CORD collective” has often been cheered for addressing challenges together. Additionally, EM educators should not shun potential solutions, even if they disagree.

### Program and Clerkship Leadership

Given current workforce projections, it is important for residency program leaders to be introspective regarding the short- and long-term plans for their residencies. It may be useful for leaders at established or planned programs to answer the key questions listed in Table 1 privately, or maybe even publicly. Residency leaders should also welcome questions from applicants regarding the workforce, the program’s Match and SOAP record, and the service-to-learning balance.

Clerkship directors and EM specialty advisors should provide robust education to prospective EM residency applicants regarding the workforce and the 2022 Match, and they should help students craft respectful but probing questions for residency interviews to tease out a variety of issues, including the potential influence of business interests on resident education.

### Professional Organizations

Professional organizations have and will continue to play a significant role in EM’s response to the predicted workforce crisis because they wield powers that individuals, residency programs, and departments do not have. These organizations can elevate the standards required of EM residency programs, formulate consensus statements, research best practices, and even lobby for potential solutions.

A working group with representatives from CORD, ACEP, American Academy of Emergency Medicine (AAEM), the Society for Academic Emergency Medicine (SAEM), American College of Osteopathic Emergency Physicians, Association of Academic Chairs of Emergency Medicine (AACEM), Emergency Medicine Residents’ Association, AAEM Resident and Student Association, and SAEM Residents and Medical Students convened to strategize solutions to the predicted oversupply of EPs.

In March, these organizations published a joint statement addressing the 2022 Match, highlighting the growth in EM residency positions, the utility of continuing to study workforce dynamics, and the importance of working together toward solutions.^11^ In April, a draft of the group’s recommended updates to the ACGME EM program requirements was distributed.^12^ If adopted, these more stringent and often research-based EM residency program guidelines would ensure higher quality training and could slow the growth of EM residency positions as new, expanding, or even current programs may not be able to meet the new bar. Hopefully EM organizations will continue to jointly explore interventions that could improve training while addressing the workforce.

Emergency medicine organizations could even more directly address the supply side of the workforce by developing guidelines aimed at reducing program creation and complement increases, except in exceptional circumstances. While these guidelines would not be enforceable, they would still tighten the reins on workforce expansion; for example, organizations ignoring such guidelines would have to share their reasoning when interviewing prospective residents. Table 2 lists potential guidelines that could be adopted individually or jointly by national EM organizations.

CORD appears to be in a bind when it comes to making statements or guidelines regarding the Match and the EM workforce. To some extent, both SAEM and AACEM find themselves in similar positions. Although not explicitly stated in its mission, vision, and purpose statements,^13^ one of CORD’s guiding principles is avoiding any action that could harm an EM residency program. Therefore, CORD’s primary mechanism for addressing the supply side of the workforce has been via reinforcing academic standards, a very principled and egalitarian approach. While CORD takes a “port in the storm” approach, it should be noted that during a hurricane, ships are sometimes safer at sea.^14^ CORD’s attempts to address the predicted workforce crisis could be more impactful if it took the approach of balancing the needs of the specialty with the needs of individual programs. Additionally, because the over-supply of EM residency positions was created by CORD’s member programs, CORD may be in a unique position to influence programs to slow or even reverse this growth.

In addition to making guidelines and advocating to strengthen residency standards, EM professional organizations can address the supply side of the workforce in a variety of other ways as well. Table 3 expands on strategies professional organizations could employ to address the supply of EPs.

## CONCLUSION

Educators in EM have the opportunity to help guide the specialty through an inevitable inflection point. Proactively addressing the supply side of the EM workforce risks creating near-term pain points, including a temporary decrease in EM residency applicants. Simultaneously, however, having uncomfortable discussions, exploring potential solutions, and making difficult decisions now could strengthen and fortify the specialty for decades.

## Figures and Tables

**Table 1:** Key questions for program directors.

Does the program have the “right” complement of residents given workforce projections? Even if the program could provide superb education to more residents, how would expanding the residency benefit EM given workforce projections? Are there legitimate arguments for decreasing the number of residents in the program? Does the program provide the right balance of service to learning? Even if a planned new program would provide superb education, how would that program benefit EM given workforce projections? What proportion of the motivation for expanding a current program or developing a new program is financial gain?

*EM*, emergency medicine.

**Table 2:** Potential guidelines from national EM organizations addressing the supply side of the EM workforce.

With rare exception, we discourage the creation of additional EM residency positions, either by program expansion or the development of new programs. We recommend any new EM residency positions be specifically designed to (1) recruit applicants who are underrepresented in medicine into EM, (2) have residents work in EM “deserts” after graduation, or (3) both. We recommend that each new and expanding EM residency program craft a statement describing its reasoning for creating new EM residency positions given recent workforce projections. We recommend that all programs evaluate their complement of residents and consider whether a complement reduction would be feasible and good for the specialty. We recommend that programs who do not fill in the Match (not the SOAP) for consecutive years consider a complement decrease. We recommend that programs not take applicants in the SOAP who had no intent to match into EM, as they have not thoroughly explored the specialty and do not have SLOEs. We recommend that clerkships educate EM-interested medical students regarding EM workforce projections, including events leading to the projected EP surplus and steps the EM community is taking to address the issue. We recommend that applicants to EM residency programs strongly consider potential factors that could skew the balance between education and service, including lack of ancillary services, for-profit hospital ownership, and corporate influences in the emergency department. We recommend that applicants to EM residency programs strongly consider potential risks and benefits of applying to programs that do not regularly fill in the Match. We recommend that health systems not push departments to open or expand EM residency programs in an attempt to increase their GME cap on resident trainees. We recommend that health systems not punish departments for choosing not to open or expand EM residencies, even if there is a financial argument to do so.

*EM*, emergency medicine; *SOAP*, Supplemental Offer and Acceptance Program; SLOE, Standard Letter of Evaluation; *EP*, emergency physician; *GME*, graduate medical education.

**Table 3:** Strategies professional organizations could employ to address the supply of emergency physicians.

Advocating to strengthen EM residency standards; Developing consensus statements and guidelines to limit or reverse the growth of EM residency positions; Providing frequent communications to members explaining the organization’s approach to the EP oversupply and detailing actions planned or already taken; Making time during academic or scientific assemblies for information sharing, question and answer sessions, and open and honest discussion; Tasking committees to brainstorm solutions; Sponsoring research evaluating proposed solutions; and Continuing to advise the ACGME EM Residency Review Committee (RRC), including Recommending that the RRC deny accreditation of programs with red flags instead of granting accreditation with warning, and Highlighting the downsides of EM residency expansion and the danger of the disproportionate growth of programs in regions already heavily populated by EM residencies.

*EM*, emergency medicine; *EP*, emergency physician; *ACGME*, Accreditation Council for Graduate Medical Education.
